# Why Don’t All Individuals Who Undergo Dura Mater/Arachnoid Puncture Develop Postdural Puncture Headache?

**DOI:** 10.5812/kowsar.22287523.3616

**Published:** 2012-01-01

**Authors:** Marcelo M. Valenca, Jane A. Amorim, Tiago P. Moura

**Affiliations:** 1 Neurology and Neurosurgery Unit, Department of Neuropsychiatry, Federal University of Pernambuco, Recife, Brazil

**Keywords:** Post-Dural Puncture Headache, Anesthesia, Spinal, Dura Mater

Dear Editor,

The physiopathology of postdural puncture headache (PDPH) is most likely multifaceted (1, 2). Understanding the mechanisms through which some subjects develop PDPH may help in the development of PDPH-prevention strategies. In addition, identifying risk factors in susceptible individuals may help develop forms of anesthesia other than spinal anesthesia for preventing dura mater/arachnoid perforation and, subsequently, PDPH (3). Clearly, the main reason and the sine qua non for accounting for PDPH is an excessive loss of cerebrospinal fluid (CSF) from the subarachnoid space after the puncture of both dura mater and arachnoid. 

**Figure 1.**
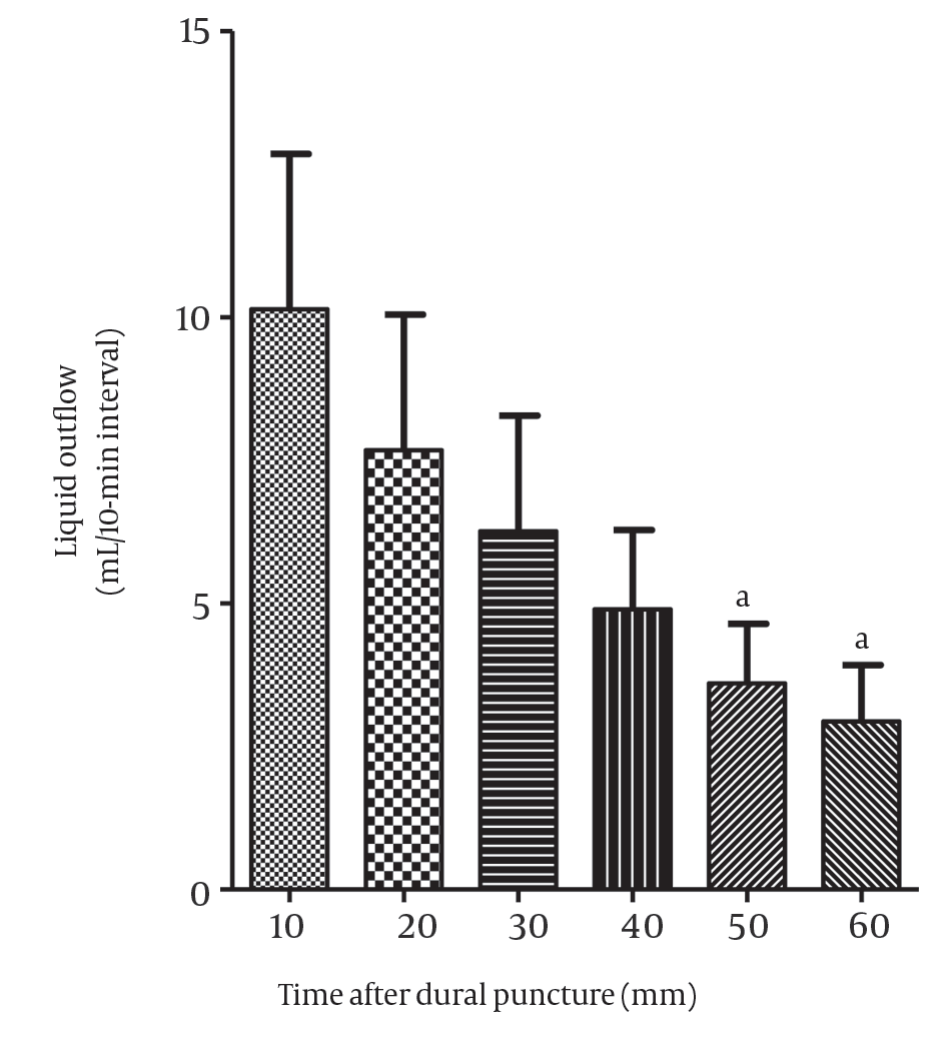
Volume (mL) of Liquid Outflow After Dura Mater Puncture Using a Model of Dural Sac With the Help of an Acrylic Column With Human Cadaver Dural Attachment Mimicking an In vivo Situation (40 cm H2O Pressure at the Level of Puncture, n = 23). ^a^ P < 0.05 vs. the first 10 min interval (0-10 min), Kruskal-Wallis and Dunn’s multiple comparison test

However, it is unclear why all individuals who suffer from dura mater/arachnoid puncture do not develop PDPH. Additionally, it is unclear why women are more susceptible than men to PDPH (3, 4). Recently, we demonstrated that, after perforating human cadaver dura mater using dural sac model with the help of an acrylic column with a dural attachment mimicking an in vivo scenario (40 cm H2O pressure at the level of puncture), the liquid outflow was higher using female-derived dura mater fragments than male-derived fragments. In addition, after perforation of the dura mater, the initial liquid outflow was highly variable between dura mater specimens (3.7 ± 5.0 [SD] mL/10 min, median 2.2 mL/10 min; minimum 0 mL/10 min, maximum 18 mL/10 min, n = 17; using a 27-gauge Quincke needle), even when different fragments of the same cadaver donor were tested (e.g. 0 mL/10 min, 2.5 mL/10 min, 6.2 mL/10 min and 14 mL/10 min of liquid outflow from each of the 4 distinct tested dural fragments, respectively; 52-year-old female). These findings explain why only some subjects develop PDPH. Another noteworthy point is that during the 60-minute experiment, the liquid outflow decreased with time (Figure 1), in some of the perforated fragments a spontaneous arrest was observed. In 5 of the 17 dura mater-tested fragments, we did not observe any loss of liquid after the perforation of the dura mater by the insertion and removal of the needle. This demonstrated that the dura mater has an intrinsic elastic mechanism that enables it to restore or occlude the orifice produced by the needle and also that this characteristic is variable when considering different tested specimens. In our series(5), women were at greater risk of PDPH than were men (10.6% vs. 2.9%). This result was similar to the findings of Wu et al.’s (4) meta-analysis of nonpregnant women, which showed that the risk of PDPH was twice as great as that of men, irrespective of age, needle caliber, or design of the bevel. In addition to the abovementioned aspects of dura mater CSF loss, possible explanations lie in the physiological, anatomical, social, and behavioral characteristics specific to women, as well as their perception of pain (3-6). High levels of estrogen in women seemed to interfere with the tone of the cerebral vessels, probably increasing the vascular distension response to CSF hypotension (7). Furthermore, women seemed to process the nociceptive information differently than men, exhibiting greater sensitivity to painful stimulation, thus facilitating the central sensibilization process as shown in neuroimaging studies (8). Another aspect to be considered is the erroneous idea that the dura mater fibers are aligned longitudinally. Indeed, studies have demonstrated that these fibers run in different directions, with heterogeneous interactions between them (9). In an evaluation of multiple predictors of PDPH resulting from the use of 22-G, 25-G, and 26-G cutting needles, Lybercker et al. (10) found that the perpendicular orientation of the bevel was a predictive factor, just as we found in our study (10). In their meta-analysis, Richman et al. (11) evaluated the influence of Quincke and Tuohy needles (bevel cutting) on the incidence of PDPH in adult patients. They demonstrated that, compared to a perpendicular orientation, a bevel orientation parallel to the long axis of the spinal column significantly lowered the incidence of PDPH (10.9% vs. 25.8%; odds ratio of 0.29 [95% CI = 0.17-0.50]) (11). In this regard, it has been postulated that the arachnoid may be at least as important as the dura mater, and indeed perhaps more so, in the genesis of PDPH. In an experimental study, Kempen and Moeck found that, when a puncture was made with a parallel orientation of the bevel, the layers of dura mater and arachnoid overlapped and that this overlap could reduce CSF leakage (12). Zetlaoui (13) suggested that variations in the diameter of the puncture hole in the dura mater are due to the movements and pricks made in the dural sac. When the bevel is parallel to the neural axis, the prick, which opens up when the patient sits up or stands, tends to close. With the bevel perpendicular, the hole is enlarged and there is a subsequent loss of CSF (13). As for the angle of needle insertion, an in vitro study, using a model of human dura mater, demonstrated a smaller loss of CSF when the needle was inserted using the paramedian approach (0.3 ± 0.4 mL/min). In contrast, when the median approach was used, the loss of CSF was greater (3.3 ± 1.6 mL/min) (14). One possible explanation would be that the paramedian approach decreases the loss of CSF resulting from perforation of the dura mater and the arachnoid at different angles, producing a valvular mechanism that prevents a greater CSF flow to the epidural space. In a study by Mosaffa and colleagues (15), no significant association between the angle of approach and the incidence of PDPH was found. Imbelloni et al. also did not find any significant association between the needle’s angle of approach and the incidence of PDPH in a study with Quincke needles and a cutting bevel, caliber 25 G and 27 G (median 4.2% vs. paramedian 0.7%, P = 0.071, Fisher’s exact test) (16). In our sample of patients, the paramedian approach resulted in a lower incidence of PDPH, although this incidence was not statistically different than the incidence the median approach (5.5% vs. 8.6%; P = 0.17).

In conclusion, one of the reasons that not all individuals who undergo dura mater/arachnoid puncture develop postdural puncture headache is that dura mater can attenuate or even prevent CSF loss after a puncture through a dynamic phenomenon of orifice occlusion, even when fresh cadaver specimens of dura mater are tested in vitro. This characteristic varies among individuals and perhaps also between different parts of the dura mater of the same individual.
